# Implantable and transcutaneous photobiomodulation promote neuroregeneration and recovery of lost function after spinal cord injury

**DOI:** 10.1002/btm2.10674

**Published:** 2024-04-25

**Authors:** Andrew R. Stevens, Mohammed Hadis, Alice Phillips, Abhinav Thareja, Michael Milward, Antonio Belli, William Palin, David J. Davies, Zubair Ahmed

**Affiliations:** ^1^ Neuroscience and Ophthalmology Institute of Inflammation and Ageing, University of Birmingham Birmingham UK; ^2^ NIHR Surgical Reconstruction and Microbiology Research Centre University Hospitals Birmingham Birmingham UK; ^3^ Phototherapy Research Group, School of Dentistry University of Birmingham Birmingham UK; ^4^ School of Dentistry University of Birmingham Birmingham UK; ^5^ Centre for Trauma Sciences Research University of Birmingham Birmingham UK

**Keywords:** medical devices, neuroregeneration, photobiomodulation, spinal cord injury, spinal cord stimulation

## Abstract

Spinal cord injury (SCI) is a cause of profound and irreversible damage, with no effective therapy to promote functional recovery. Photobiomodulation (PBM) may provide a viable therapeutic approach using red or near‐infrared light to promote recovery after SCI by mitigating neuroinflammation and preventing neuronal apoptosis. Our current study aimed to optimize PBM dose regimens and develop and validate the efficacy of an invasive PBM delivery paradigm for SCI. Dose optimization studies were performed using a serum withdrawal model of injury in cultures of primary adult rat dorsal root ganglion neurons (DRGN). Implantable and transcutaneous PBM delivery protocols were developed and validated using cadaveric modeling. The efficacy of PBM in promoting recovery after SCI in vivo was studied in a dorsal column crush injury model of SCI in adult rats. Optimal neuroprotection in vitro was achieved between 4 and 22 mW/cm^2^. 11 mW/cm^2^ for 1 min per day (0.66 J/cm^2^) increased cell viability by 45% over 5 days (*p* <0.0001), increasing neurite outgrowth by 25% (*p* <0.01). A method for invasive application of PBM was developed using a diffusion‐tipped optogenetics fiber optic. Delivery methods for PBM were developed and validated for both invasive (iPBM) and noninvasive (transcutaneous) (tcPBM) application. iPBM and tcPBM (24 mW/cm^2^ at spinal cord, 1 min per day (1.44 J/cm^2^) up to 7 days) increased activation of regeneration‐associated protein at 3 days after SCI, increasing GAP43^+^ axons in DRGN from 18.0% (control) to 41.4% ± 10.5 (iPBM) and 45.8% ± 3.4 (tcPBM) (*p* <0.05). This corresponded to significant improvements at 6 weeks post‐injury in functional locomotor and sensory function recovery (*p* <0.01), axonal regeneration (*p* <0.01), and reduced lesion size (*p* <0.01). Our results demonstrated that PBM achieved a significant therapeutic benefit after SCI, either using iPBM or tcPBM application and can potentially be developed for clinical use in SCI patients.

**Translational Impact Statement**

Photobiomodulation (PBM) may improve recovery after spinal cord injury (SCI) yet delivery of PBM to the injured spinal cord is limited by overlying tissue when applying PBM through the skin. We show the development and use of an implantable device for application of PBM which addresses this problem. Our results demonstrated that implantable PBM improved regeneration of neurons and these doses of PBM also improved functional recovery. This device has the potential to be developed to deliver PBM aimed at improving recovery in patients with SCI.

## 1 INTRODUCTION

Spinal cord injury (SCI) is a cause of profound and lifelong disability, particularly in young people.^1^ Worldwide, there are over 900,000 new injuries every year, with each case incurring an average cost of $1.4 million.^2^, ^3^, ^4^ Such injuries, often resulting from falls, road traffic collisions, or sporting accidents, can lead to permanent disabilities including loss of motor and sensory function, neuropathic pain, and loss of bladder, bowel, and sexual function.^1^ These symptoms are often permanent due to the intrinsic inability for the central nervous system (CNS) neurons to repair after injury.^1^, ^5^ The therapeutic options to promote repair and recovery of the CNS after injury remain extremely limited.^1^, ^4^, ^5^, ^6^ Pharmacological therapies, typically targeting single receptors or pathways, have thus far provided limited benefit toward the multitude of pathophysiological mechanisms required to promote effective repair after injury.^1^, ^6^, ^7^

Photobiomodulation (PBM) is the application of light for the purposes of therapeutic benefit.^8^ PBM acts principally at a mitochondrial level, with photon absorption by the photoacceptor of the electron transport chain, cytochrome C oxidase.^9^, ^10^, ^11^, ^12^, ^13^ This results in a modulation of mitochondrial membrane potential, reduced production of reactive oxygen species, and increased availability of adenosine triphosphate.^14^, ^15^, ^16^ Via undefined mechanisms, this mitochondrial initiator process triggers multiple favorable downstream pathways mitigating apoptosis, neuronal damage, and neuroinflammation and at the same stime promoting nerve regeneration.^12^, ^14^, ^17^, ^18^, ^19^, ^20^, ^21^ Previous literature exploring therapeutic applications of PBM has established evidence for its efficacy in a variety of topical (dermatological or oral) applications, where administration of photons with metered dosing can be achieved with precision due to direct‐to‐tissue delivery.^22^, ^23^, ^24^, ^25^ Beyond this, there is growing interest in the application of PBM to pathologies of deeper anatomical structures, including the CNS,^17^, ^20^, ^26^, ^27^, ^28^ particularly as a therapy for neurotrauma such as traumatic brain injury (TBI) and SCI.^17^, ^20^, ^26^

The efficacy of PBM in improving functional and histological outcomes in rodent models of SCI has been demonstrated using transcutaneous application methods,^26^, ^29^, ^30^, ^31^, ^32^, ^33^, ^34^, ^35^, ^36^, ^37^, ^38^ showing favorable mechanisms of PBM, including attenuation of macrophage/microglia/astrocyte (M1/A1 phenoytype) polarization^34^, ^35^, ^36^, ^37^, ^39^, ^40^ and neuroinflammation through interleukin (IL‐4, IL‐6) regulation,^31^, ^32^ reducing CD68 expression,^33^ STAT3 inhibition^34^, ^36^ and mitochondrial regulation.^41^ Whilst these results are encouraging of a positive biological effect, the translational potential of PBM is limited by the challenge of delivering effective and reliable doses transcutaneously to the injured CNS in humans, primarily due to the thickness of the overlying superficial tissue. PBM offers neuroprotection in rodent models of Parkinson's disease when delivered via an implant, as well as transcranially and remotely,^42^, ^43^, ^44^, ^45^, ^46^, ^47^ and direct delivery of PBM to the CNS has been demonstrated as a feasible and safe method in a porcine SCI model.^48^, ^49^ Amidst emerging evidence, the optimum method of delivery in SCI remains unclear, yet direct delivery offers a more feasible translational prospect for effective dose delivery to target tissue in humans, though the relative efficacies of invasive and transcutaneous delivery methods are yet to be established.^43^

Consequently, the overarching aim of this study was to demonstrate that invasive delivery of PBM at optimum doses after SCI promotes neurorestoration equivalent to that achieved by transcutaneous administration. We used in vitro methods to optimize dosing parameters for neuroprotection and neurorestoration, developed methods for equivalent invasive/transcutaneous delivery of PBM in a rodent model and compared the neurorestorative effects of these delivery methods using an in vivo rat model of SCI.

## 2 METHODS

All animal procedures were licensed by the UK Home Office and approved by the University of Birmingham's Animal Welfare and Ethical Review Board (PP3851114, protocol 4). Surgeries were carried out in strict accordance with the guidlines of the UK Animals Scientific Procedures Act, 1986. Power calculations using the NC3Rs Experimental Design Assistant were used to determine sample sizes and all animals were randomly assigned to treatment groups and masked to the experimenters until analysis was completed.

### 2.1 Dorsal root ganglion neuron primary cell culture

Primary adult rat dorsal root ganglion neuron (DRGN) cultures were established as described previously.^50^, ^51^, ^52^, ^53^ Briefly, *n* = 12 adult (170–220 g) Sprague‐Dawley rats (Charles River, Margate, UK) were sacrificed by exposure to rising concentrations of CO_2_. Dorsal root ganglion (DRG) pairs (T4‐L7) were removed within 60 min of death and placed in Neurobasal‐A medium (1X) (Invitrogen, Paisley, UK) at 4–8°C. DRGs were incubated in a humidified chamber at 37°C and 5% CO_2_ for 2 h in Neurobasal‐A containing 0.125% collagenase (Sigma, Poole, UK). Samples were then triturated to dissociate DRGN, centrifuged through a 15% bovine serum albumin (BSA) density gradient to remove debris and the cell pellet resuspended in media containing soybean trypsin inhibitor (Gibco) and DNase I (Sigma). Sterile 8‐well glass chamber slides (BD Biosciences, Oxford, UK) were precoated with 100 μg/mL poly‐D‐lysine and 20 μg/mL laminin (both from Sigma) and dissociated DRGN were plated at 500 cells per well in Dulbecco's modified eagle medium (DMEM) (Invitrogen) containing 10% fetal bovine serum (FBS) and 1% penicillin–streptomycin (both from Invitrogen). Following 20–24 h incubation, the medium was replaced with DMEM and 1% pen/strep: with or without 10% FBS. To control for extraneous light exposure, plates were lightly covered with aluminum foil for incubation. To assess the effect of suppression of nonneuronal cell proliferation, cultures were performed in the presence or absence of 5‐fluoro‐2‐deoxyuridine (5‐FDU, Sigma), at a final concentration of 30 μM in culture media where included.^50^, ^54^

### 2.2 Photobiomodulation treatment for primary cell culture

PBM was delivered as single daily doses, every 20–24 h, commencing within 15 min of serum withdrawal, as described previously.^18^ A total of 660 nm light was delivered with a calibrated variable power 660 nm light emitting diode (LED) array light source (BioThor device, Thor Photobiomedicine, Amersham, UK). 8‐well plates were placed above the light source, mounted on a bespoke diffuser, to directly irradiate the base of the plate. The device was calibrated for a spectrum of voltage and current outputs, measuring the wavelength and absolute irradiance values delivered to the cell culture plane.

### 2.3 Immunocytochemistry

Cells were fixed for 10 min using 4% formaldehyde, followed by 3× phosphate‐buffered saline (PBS) washes. Cells were blocked for 10 min with 1 M PBS containing 3% BSA and 0.1% Triton‐X‐100, followed by incubation with the appropriate primary antibody for 1 h at RT, diluted in 1 M PBS containing 3% BSA. Plates were washed and incubated with secondary antibody for 1 h before mounting in Vectamount with 4′,6‐diamidino‐2‐phenylindole (DAPI) (Vector Laboratories, Peterborough, UK).

### 2.4 Microscopy and image analysis

Immunostained slides were viewed under an Axioplan 2 fluorescent microscope with images captured using a mounted Axiocam HRc camera running Axiovision Version 4.0 Software (all from Carl Zeiss, Welwyn‐Garden City, UK). Image capture and analysis were performed by an investigator blinded to the treatment conditions, as described by us previously.^55^ To determine cell viability, wells were divided into nine equal sections, and one image taken per section. Viable DRGN were identified with βIII‐tubulin and DAPI co‐staining. To measure neurite outgrowth, after immunostaining for βIII‐tubulin, images were taken of 30 randomly selected DRGN per well. The length of the longest neurite and number of branch points of the longest neurite were measured using NeuronJ (ImageJ), and the number of DRGN with neurites was recorded. For each condition, at least 180 DRGN were analyzed for neurite outgrowth parameters.

### 2.5 Cadaveric modeling

To model the in vivo transmission of 660 nm light in both invasive and noninvasive administration models, cadaveric modeling was performed. Adult Sprague‐Dawley rats (170–220 g, *n* = 6, male: female 1:1, Charles River) were sacrificed by exposure to increasing concentrations of CO_2_. After sacrifice, the procedure for dorsal column crush (DCC) was performed as described below. For all cadavers, an anterior approach was then performed with midline thoracotomy, laparotomy, and total evisceration from thoracic and abdominal compartments. An anterior approach corpectomy (T6–T10) was performed, and spinal cord divided at T5/6 and T10/11, with the intervening spinal cord removed. This approach exposed the laminectomy site from the anterior aspect. To record spectral irradiance, the fiber tip was positioned within the corpectomy site at the mid‐point of the spinal canal (in the anterior–posterior plane). For beam profiling, the camera was focused on this plane.

### 2.6 Photobiomodulation administration ex vivo

For invasive delivery of 660 nm light, a calibrated 660 nm coherent laser source (MDL‐III‐660‐FC‐800 mW, CNI Co., Ltd., Changchun, China) was used. The source was coupled to a rotary joint patch cable (FT200EMT) via a subminiature version A (SMA)‐SMA mating sleeve (ADASMAB3), coupled to the implant (both from Thor Labs). Implants were fabricated using three components: 200 μm 0.39 NA 10 mm diffusion tip catheter (CFDSB10); implant guide (OGL‐5); and phosphor bronze split mating sleeve (ADAL4‐5) (all from Thor Labs). Individual components were cleaned using 70% ethanol and allowed to air dry in a sterile cabinet. Once dry, components were fixed together using a commercially available cyanoacrylate (Loctite, Westlake, OH, USA), and allowed to cure at room temperature under ambient lighting for 24 h. After curing, implants were sterilized using ultraviolet light in a UVP CL‐3000 (Analytik‐Jena, Jena, Germany) cabinet, for 3× 10 min with 120° rotation between each cycle.

For transcutaneous delivery, two devices were trialed: an MDL‐III 800 mW calibrated source (as above), and an LX2 75 mW 660 nm Laser Therapy System with manufacturer‐supplied probe (Thor Labs). In both cases, the device was placed in direct contact with skin overlying the injury site and orientated toward the laminectomy site. As described below, for in vivo experiments MDL‐III 800 mW was used.

### 2.7 Beam characterization

Beam characterization was performed with an SP620 camera and BeamGage Standard software (6.17), (both Ophir Photonics, Darmstadt, Germany). Neutral density filters were used where required to optimize characterization within the tolerances of the SP620 to avoid sensor saturation (ND1/2, Ophir Photonics). Linear calibration was performed, and ambient light calibration was performed using the Ultracal function of the integrated Beamgage software. D4σ values were derived from beam diameters using the integrated Autoaperture function. For direct beam profiling the SP620 camera focused onto a target screen (LMR2/M, Thor Labs) at a specified distance from the emitting fiber.

Power calibration values were derived through two methods. For spectral irradiance, spectrometry was acquired with a USB4000 spectrometer coupled to a 200 μm fiber with a CC‐3‐UV‐T cosine corrector for an inlet aperture of 400 μm (all from OceanOptics, Ostfildern, Germany). The spectrometer was calibrated using a standard halogen light source (DH‐2000, OceanOptics). Spectral irradiance was calculated via area‐under‐the‐curve integration calculation (Microsoft Excel, Microsoft, USA). Where aperture or sensitivity range of spectrometer system were not compatible with measurement, power values were acquired with PD300R‐3 W power meter (10 mm aperture, used with manufacturer‐supplied removable filter) and StarLab 3.0 software (all from Ophir Photonics).

### 2.8 Thermal imaging

For thermal imaging of the temperature changes associated with light administration, an AX5 (Teledyne forward‐looking infrared (FLIR) LLC, Oregon, USA) camera was focused onto the tissue in contact with the highest irradiance from a given source. Image capture and thermal profiling was performed using FLIR software ResearchIR (Teledyne FLIR). Animal tissue was assigned an emissivity of 0.95, probes were assigned an emissivity of 0.90.

### 2.9 In vivo dorsal column crush

Dorsal column injury model was performed as described elsewhere.^50^, ^53^, ^56^, ^57^, ^58^ Power calculations using values from previously published data supported group sizes of 2–4 and hence 4 rats/group were used. Briefly, adult Sprague‐Dawley rats (190–250 g, *n* = 50, male: female 1:1, Charles River) were anesthetized using inhalational anesthesia (5% isofluorane with 1.8 L/min O_2_) and a partial laminectomy was performed at thoracic level 8 (T8) before bilaterally crushing the dorsal columns using calibrated watchmakers forceps. For invasively delivered PBM, the surgical wound was closed with inclusion of an implant (described below). The tip of the catheter was positioned directly overlying the injury site and implants were secured using heat‐sterilized surgical glue around the laminectomy site without contact with the spinal cord. The implant was further secured to the dorsal muscle and skin using a 4‐0 vicryl suture, with 5 mm of the phosphor bronze mating sleeve remaining external for intermittent connection to deliver PBM treatments. For “sham” procedures, all steps were completed with the exception of the DCC or implant. Pre‐operative and post‐operative analgesia was given (intraperitoneal buprenorphine) as standard and as recommended by the veterinary surgeon. For all procedures using implants, visual confirmation was made during dissection after termination that the implant remained intact and correctly positioned.

### 2.10 Photobiomodulation treatment in vivo

For invasive delivery of 660 nm light, implants were fabricated as described above. For treatments, a calibrated 660 nm coherent laser source (MDL‐III‐660‐FC‐800 mW, CNI Co., Ltd.) was used. The source was coupled to a rotary joint patch cable (FT200EMT) via an SMA‐SMA mating sleeve (ADASMAB3), coupled to the implant (both from Thor Labs).

For transcutaneous delivery an MDL‐III 800 mW calibrated source (as above) coupled to a custom fiber bundle (FG550UEC, Thor Labs). The diffuser tip of the probe was placed in direct contact with skin overlying the injury site (immediately lateral to the skin incision, and in line with T8 in the rostro‐caudal plane) and orientated toward the laminectomy site.

The first treatment was given 15 min post‐injury whilst the animal remained anesthetized. Subsequent doses were given every 20–24 h, for either 3 days or 7 days. Probe positioning or implant connection to the external source was achieved by mild manual restraint. All doses (via implant or transcutaneous methods) were calibrated to deliver 24.42 mW/cm^2^ at the level of the spinal cord as developed and validated above.

### 2.11 Functional assessments

All functional assessments were recorded in duplicate by two blinded investigators. In the event of discrepancy, a mean of the two scores was used. *Horizontal ladder crossing* ^50^, ^56^: prior to surgery, rats were habituated and trained to cross the horizontal ladder. The ladder is composed of two clear Perspex sheets, with randomly distributed “rungs” (3 mm plastic rods) inserted to create a horizontal ladder. On each trial, the ladder rungs were rearranged to prevent a learning effect. Three attempts were given to cross the ladder, with no time limit imposed. Each step with a hindlimb was recorded as a “step” (successful landing and push‐off of each rung) or a “slip” (a missed step, unstable landing, or failed push‐off). For analysis, a step: slip ratio was used as a mean of three repetitions per week. *Tape sensing and removal test* ^50^, ^56^: a 1 cm × 1 cm square of laboratory adhesive tape was adhered to the palmar surface of the left hind paw, and the animal was returned to a cage with a plastic floor. Animals were observed for 90 s, with “sensing” recorded after localization to the paw with intent to remove the tape, and “removal” recorded after successful removal of the entire piece of tape. Failure to sense or remove the tape after a full 90 s was recorded as a failure and the piece of tape was removed by an investigator. Trials were performed on one occasion per animal, weekly for 6 weeks, after habituation prior to the injury.

### 2.12 Immunohistochemistry

At 3 days or 6 weeks post‐injury, animals were sacrificed by exposure to increasing concentrations of CO_2_ with confirmation of permanent cessation of the circulation. Immediately after death, animals were perfused with 4% paraformaldehyde by exposure of the heart and cannulation of the left ventricle. After fixation, tissues for immunohistochemistry were dissected: a 1.5 cm segment of spinal cord centered on T8; 0.5 cm lengths of spinal cord rostral (T7) and caudal (T9) to the injury site segment (as described previously^53^, ^57^); and L4/5 pairs of dorsal root ganglia (DRG). Tissues were post‐fixed in 4% paraformaldehyde for 2 h and washed in PBS three times. Cryoprotection was achieved using increasing concentrations of sucrose solution (10%, 20%, 30%) before embedding in optimal cutting temperature (OCT) compound and stored at −80°C until use.

Sagittal sections of T8 lesion sites were taken in 15 μm slices using a cryostat (OTF, Bright Instruments, UK). The entire tissue sample was cut and numbered, to allow the central section (at the lesion site epicenter) to be selected for analysis. Tissue sections were adhered to charged slides (SuperFrost^+^, Fisher Scientific, USA) and stored at −20°C until use. After defrosting at room temperature, slides were washed in 1 M PBS and permeabilized in a solution containing 0.1% Triton‐X‐100. After further washing in 1 M PBS, tissue sections were blocked in a solution containing 0.5% BSA and 0.1% Triton‐X‐100. Sections were then incubated with primary antibody diluted in a solution containing 0.5% BSA and 0.01% Tween‐20. Incubation was performed overnight at 4°C. After incubation, slides were washed in PBS before incubation with secondary antibody for 1 h at room temperature. Slides were washed before mounting coverslips with Vectamount with DAPI (Vector Laboratories). Antibody clones used are given in Supplementary Table 1.

### 2.13 Microscopy and image analysis

Immunostained slides were viewed under a fluorescent microscope with images captured using a mounted Axiocam HRc camera (all from Carl Zeiss). Image capture and analysis were performed by an investigator blinded to the treatment conditions. After image capture (taken at 20× magnification, approximately 50–200 images per tissue section), composite images were rendered using Photoshop (Adobe, Atlanta, GA, USA) via the Photomerge function. Image processing and axonal counts were performed using ImageJ (National Institutes of Health, Bethesda, Maryland, USA).

### 2.14 Quantification of regenerating axons

For axon quantification, in our previous experience, cholera B toxin tracer methods are not effective in identification of regenerating axons in rat models.^58^ As such, GAP43 immunoreactivity was used in sagittal midline T8 injury site sections as previously described.^50^, ^53^, ^56^, ^57^, ^58^ Using the sagittal slices from the center of the injury site at the midline, the entire 15 mm section was imaged at 20× magnification, with a composite image rendered using Photoshop. Using ImageJ, the rostrocaudal center of the lesion was identified based on laminin co‐staining, and markers were placed at intervals rostral and caudal to the epicenter. GAP43^+^ immunoreactivity in rostro‐caudally orientated linear streaks were counted where they intersected a line drawn perpendicular to each distance marker, to the requisite depth of the injury. As these are ascending neurons, these axonal counts for each distance are presented as a percentage of the maximum number of GAP43^+^ axons identified at the furthest observed distance (caudal) to the lesion site.

### 2.15 Quantification of GAP43 ^+^ DRGN

For DRG sections, five images were taken from each specimen at 10× magnification. An investigator blinded to the treatment conditions took images from the maximal regions of GAP43 immunoreactivity including one image from each of the DRG (L4/5 bilateral DRG). After image acquisition, background removal and autothresholding was performed in ImageJ for both GAP43 (Alexa Fluor 488) and NF200 (Alexa Fluor 594). Cell counts for each image were performed and expressed as a ratio.

### 2.16 Evaluation of glial scar

Using the central sagittal slices (2 × 15 μm slices per animal) of the injury site, laminin staining was evaluated using ImageJ.^58^ After image scaling, the extent of laminin staining and cavitation were traced manually. Where cavitation extended beyond laminin immunoreactivity, this area was included. Within this region of interest, after background removal and autothresholding, average density was measured within ImageJ.

### 2.17 Statistical analysis

Statistical analysis was performed using Prism 9.2.0 (GraphPad, California, USA). Shapiro–Wilk analysis was performed to test for normal distribution. For nonparametric data: Wilcoxon signed‐rank test was used for two‐group comparison; for comparison between more than two groups, Friedman test was used. For parametric data: unpaired t‐test was used for comparison between two groups; for comparison between more than two groups, row‐matching one‐way ANOVA was used with Geisser–Greenhouse correction. Functional outcomes were assessed using R package (www.r-project.org) This is described in detail elsewhere.^56^ Tape sensing and removal tests were analyzed by linear mixed modeling using *pbkrtest* and the Kenward–Roger method. Binomial generalized linear mixed models were used to assess horizontal ladder crossing tests, with models fitted using *lme4* with *glmer* function, with parametric bootstrap to calculate *p*‐value.

Graphs and figures utilize the following symbols to denote or identify: *n*s = *p* >0.05; * = *p* <0.05; ** = *p* <0.01; *** = *p* <0.001; *** = *p* <0.0001; # = lesion site; dashed boxes = inset boundaries; arrows indicate GAP43^+^ axons. Graphs show mean ± standard error of the mean (SEM) throughout the manuscript.

## 3 RESULTS

### 3.1 In vitro dose delivery calibration

**FIGURE 1:**
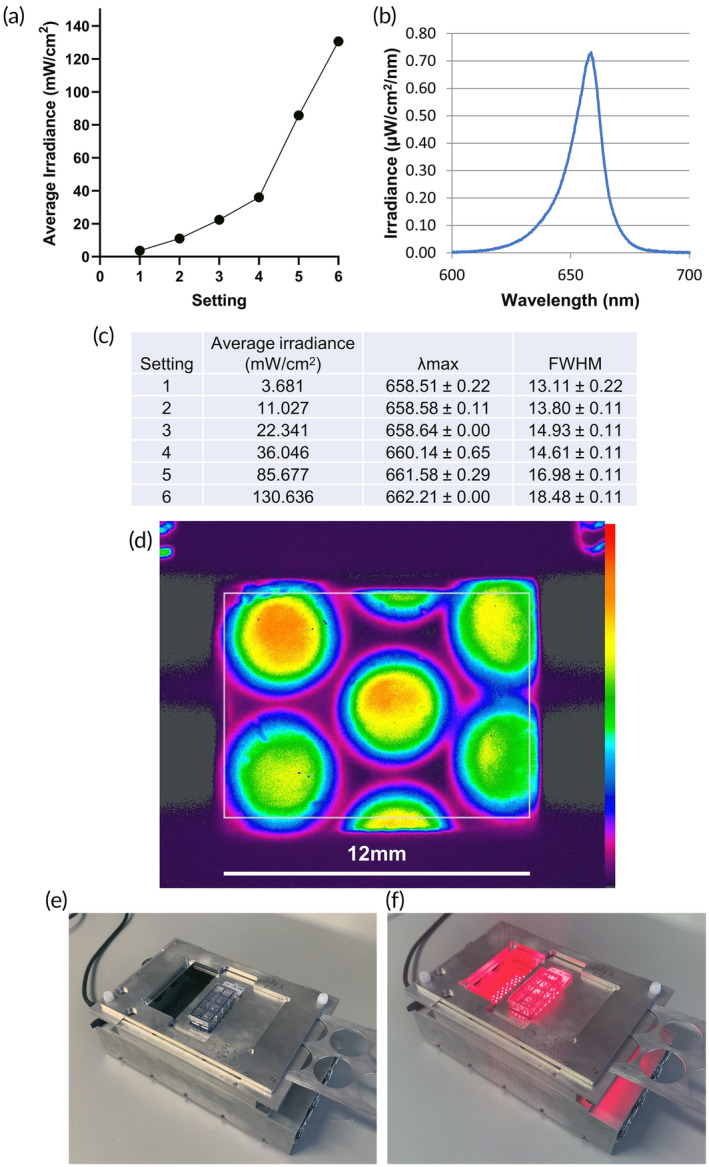
Profiling of a device for the metered delivery of PBM to DRGN in culture. (a) Average irradiance of the 6 settings used for the variable power 660 nm LED array light source (BioThor device, Thor Photobiomedicine); (b) Spectral irradiance for setting 2; (c) Average irradiance, λmax and FWHM (full‐width half maximum) values for each setting profiled, error show as SD; (d) Beam profile through single well of 8 well plate and plate diffuser, relative intensity color bar to right (OSI Rainbow, maximum intensity top; minimum intensity bottom). (e) LED array in OFF mode (sham PBM delivery) and (f) in ON mode (PBM delivery).

A custom PBM delivery device was calibrated to deliver a range of irradiances to DRGN in culture in an 8‐well glass chamber slide (Figure 1). The calibrated average irradiance values were 3.7, 11.0, 22.3, 36.0, 85.7, and 130.6 mW/cm^2^ (Figure 1a, c), with wavelength ranging from 658.5 to 662.2 (Figure 1b, c) averaged across the distribution in each well (Figure 1d). Slides were either exposed to (a) control therapy with background broadband laboratory light (Figure 1e) or (b) 660 nm PBM at a given setting (Figure 1f).

### 3.2 PBM is neuroprotective and neuritogenic in vitro

**FIGURE 2:**
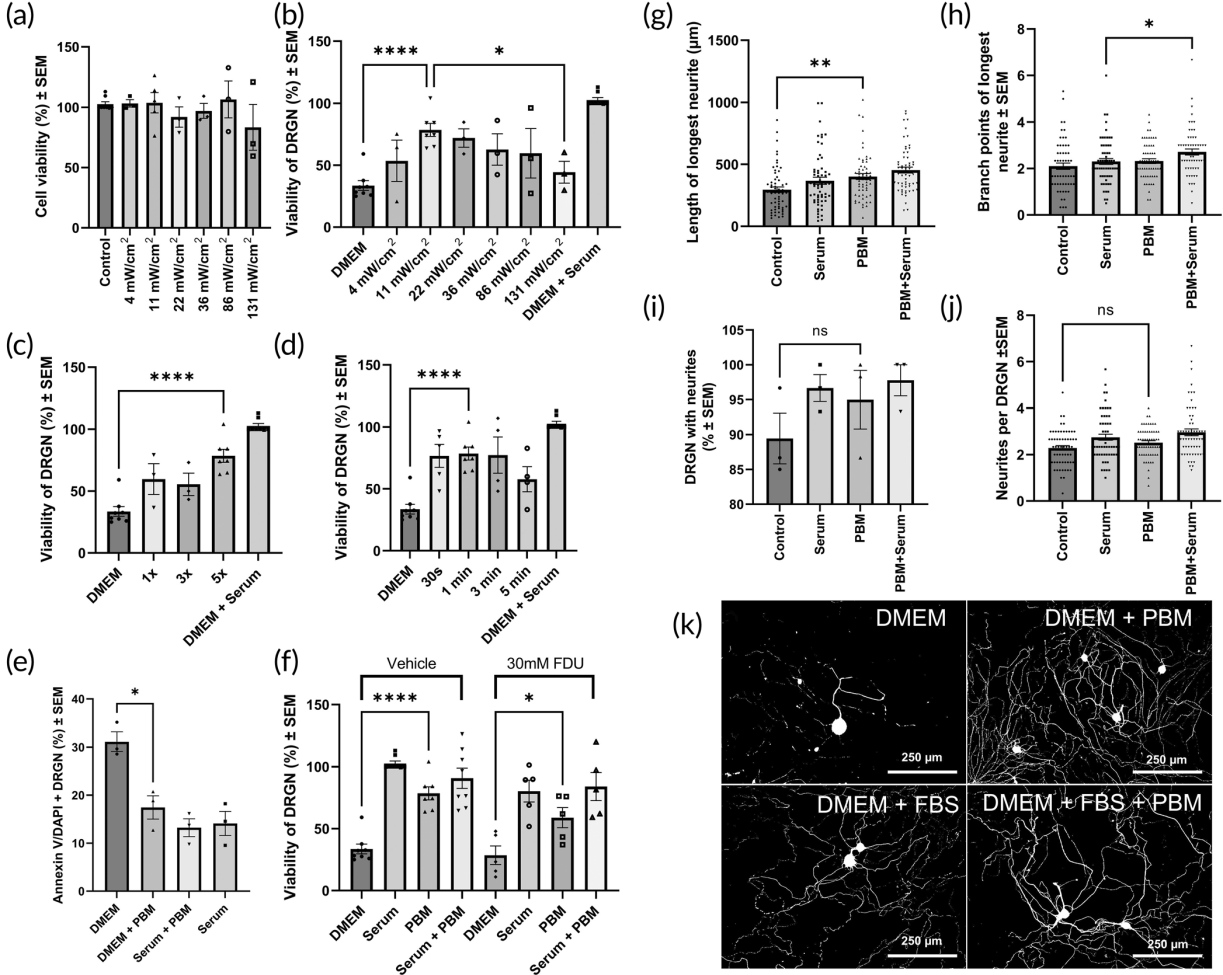
11 mW/cm^2^ PBM for 1 min per 24 h improves DRGN viability by 62% at 5 days in a serum withdrawal model of DRGN culture. (a) Samples cultured with DMEM +10% FBS for 120 h received daily exposure to PBM at six average irradiance values for 1 min per 24 h. *n* = 3. (b) Optimum viability in serum withdrawal cultures was observed in 11 mW/cm^2^ samples, with 78.6% (± 5.3) DRGN viability at 5 days. *n* = 3. (c) 11 mW/cm^2^ PBM for 1 min per 24 h on 1, 3, and 5 consecutive days of a 5 day serum withdrawal model DRGN culture. (d) 30 s to 3 min exposure daily achieves increase in DRGN viability at 5 days. (e) Annexin V + DAPI staining identified reduction in dead cells with PBM treatment at 60 h (3× PBM treatments). Viability of DRGN after 5 days increased with 5 daily PBM treatments versus 1 or 3. All cell cultures removed from incubator for 15 min per day, and 1× and 3× groups received ambient light on subsequent days after cessation of PBM. One aberrant result observed in 3× group, and pilot results presented (*n* = 2). (f) 11 mW/cm^2^ PBM for 1 min per 24 h is not significantly affected by 5‐FDU mediated suppression of nonneuronal cell proliferation. (g) Length of the longest neurite, *n* = 120 neurites from *n* = 3. (h) Number of branch points of the longest neurite. *n* = 120 neurites from *n* = 3. (i) Percentage of DRGN with neurites at 5 days. *n* = 3. (j) Number of neurites per DRGN at 5 days. *n* = 120 DRGN from *n* = 3. (*k*) βIII tubulin staining (in binary) in each condition.

The range of PBM average irradiances were used to determine the optimum value for improving DRGN cell viability in culture in a serum withdrawal model of DRGN death.^50^ DRGN were allowed to habituate in the presence of FBS for 24 h, before commencing a 5‐day culture regime either with DMEM + Serum (i.e., FBS) or DMEM alone. Differing parameters of PBM were delivered daily (every 20–24 h). First, PBM was delivered in the range of average irradiance values to cells cultured with serum, to assess the effect of PBM on DRGN in vitro in a “non‐injury” state (Figure 2a). Samples cultured with DMEM +10% FBS for 120 h received daily exposure to PBM at six average irradiance values for 1 min per 24 h. No discernible detrimental effects on DRGN survival were observed across this range.

Optimum DRGN viability after 5 days was observed with administration of 11 mW/cm^2^, associated with an increase in cell viability of 45% compared with DMEM (plus ambient light) (****) (Figure 2b). Viability of DRGN at 120 h post serum withdrawal (DMEM alone) was 33.6% ± 3.9 in comparison with continued culture with 10% FBS (100% ± 1.9). Six average irradiance values were utilized for 1 min administered every 20–24 h, with control samples receiving 1 min exposure to ambient light. Optimum viability in serum withdrawal cultures was observed in 11 mW/cm^2^ samples, with 78.6% (± 5.3) DRGN viability at 5 days. A discernible trend is shown across the irradiances, with increasing efficacy which peaks at 11 mW/cm^2^ and subsequently falls with rising average irradiance values, with 11 mW/cm^2^ demonstrating a statistically significant difference from 131 mW/cm^2^ (*).

The effect of cumulative daily exposure to 1 min PBM 11 mW/cm^2^ is shown in Figure 2c. Treatments on day 1 post‐serum withdrawal (1×) and on days 1–3 (3×) did not reach statistical significance over control when cultured for 5 days, only reached by the daily treatment regimen over the full culture period (5×). Optimal time exposure effects on cell viability were tested using 11 mW/cm^2^. All culture plates were exposed to daily 15 min intervals outside the incubator (shielded from ambient light), during which time they received either a duration (according to variable stated) of PBM therapy or 1 min ambient light exposure (Figure 2d). A plateau of broadly equivalent responses was demonstrated between 30 s (0.33 J/cm^2^) and 3 min (1.98 J/cm^2^), with a reduction in efficacy seen with 5 min (3.3 J/cm^2^) exposure.

To confirm the survival effect of PBM on averting apoptosis in DRGN, Annexin‐V live culture assay was used to determine the proportion of dead cells (from apoptosis) after 72 h in culture, showing that PBM significantly reduced this proportion of the remaining cell population (Figure 2e). The effect of culture with and without suppression of nonneuronal cell proliferation (with addition of 30 mM 5‐fluoro‐2‐deoxyuridine (5‐FDU) to the culture media^50^, ^54^) was assessed (Figure 2f), and the therapeutic effect occurred independently of proliferative suppression.

Using the optimum PBM conditions for promoting DRGN survival in a serum withdrawal model in vitro (1 min per day, 11 mW/cm^2^, 0.66 J/cm^2^), the effect of 660 nm PBM on neurite outgrowth was investigated. PBM was demonstrated to increase the length of the longest neurite (Figure 2g, k). Length of the longest neurite in control serum withdrawal samples was 295.3 μm (± 22.7) and 367.9 μm (± 28.61) in samples cultured with DMEM +10% FBS with daily ambient light exposure. The length of longest neurite in serum withdrawal samples with PBM was 402.0 μm (± 23.9) and with serum + PBM was 453.8 μm (± 23.7).

There were no effects of PBM after serum withdrawal on branching, number of DRGN with neurites, or number of neurites per DRGN (Figure 2h–j). The number of branch points of the longest neurite increased significantly with the application of daily PBM at 11 mW/cm^2^ for 1 min per 24 h in samples cultured with DMEM +10% FBS. However, there was no significant difference at the level of in the percentage of DRGN with neurites at 5 days, nor in the number of neurites per DRGN at 5 days.

### 3.3 Invasive delivery of PBM to the injured rat spinal cord

**FIGURE 3:**
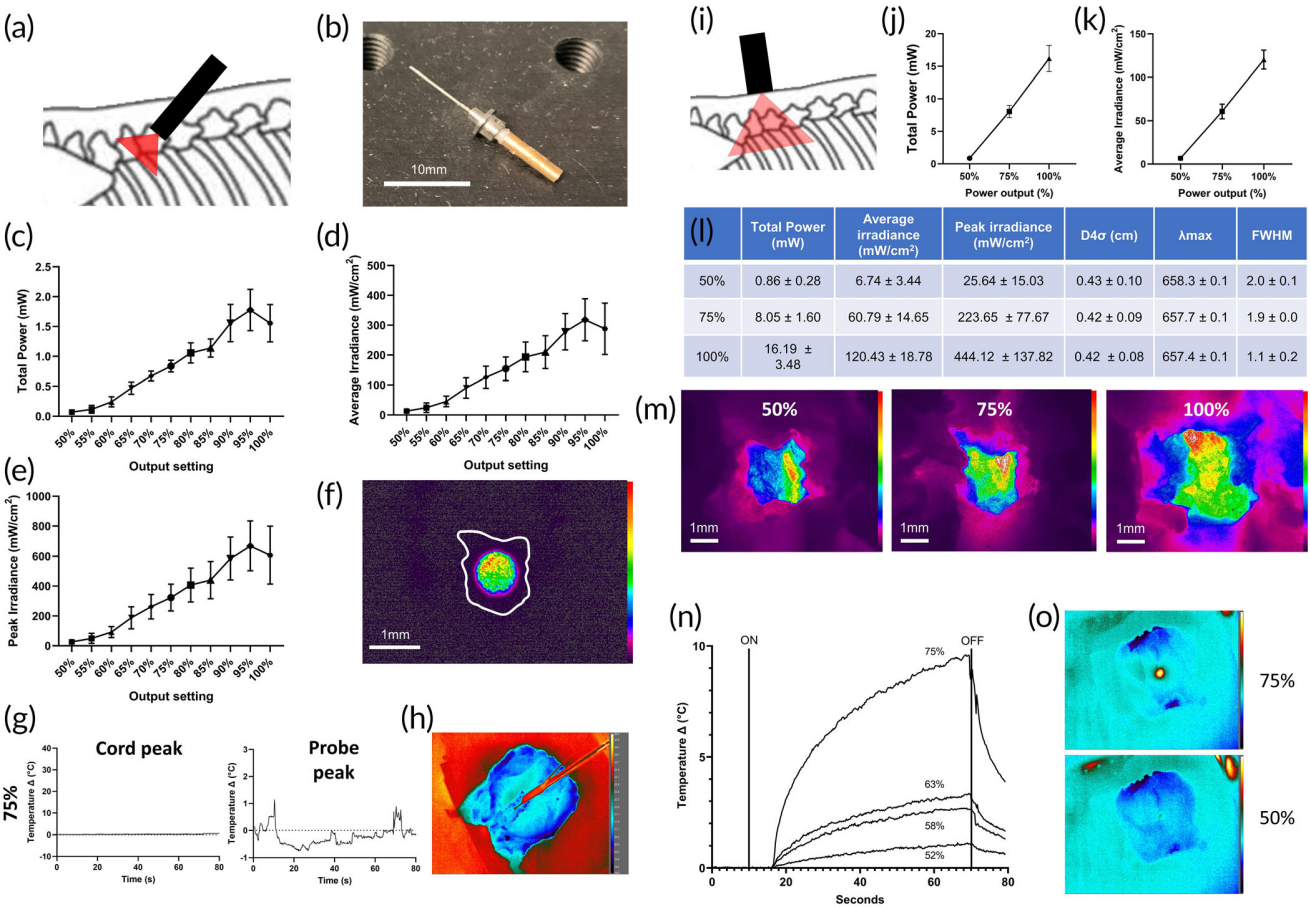
PBM device parameters. (a) Concept of direct PBM via implant. (b) An Implantable PBM device—200 μm 0.39 NA 10 mm diffusion tip catheter (CFDSB10) with implant guide (OGL‐5) (coupled to 800 mW MD‐III laser source, not pictured). (c)–(f) Beam characterization outputs focussed at the level of dorsal spinal cord in a cadaveric model of post‐procedure adult Sprague‐Dawley rat at varying power output values of the MD‐III laser (50%–100% output); (c) total power; (d) average irradiance; (e) peak irradiance. *n* = 3. (f) beam profile of implant output at 55% power output setting, silhouette of laminectomy site circumscribed in white. Relative intensity color bar to right (OSI Rainbow, maximum intensity top; minimum intensity bottom). (g) Safe levels of tissue heating are demonstrated by thermal imaging at 75% power output settings delivered directly to cadaveric spinal cord via implant. 100% power output resulted in unacceptable levels of tissue heating. OFF (0 s) and ON (60 s) temperatures for 75% power output settings, in an implant placed directly over the ventral aspect of the thoracic spinal cord after anterior dissection and corpectomy. *n* = 3. (h) FLIR imaging of ventral view of thorax (with spinal cord exposed via T6‐10 corpectomy) after 60 s exposure to 75% power output via implant directly ventral to spinal cord. (i) Concept of tcPBM via direct‐to‐skin laser source. (j) Beam profiling of transcutaneous delivery of 63 mW (50%), 427 mW (75%) and 801 mW (100%) laser power, with (j) total power; (k) average irradiance; (l) table of values for each variable given; (m) ventral view of laminectomy at the position of the dorsal column, relative intensity color bar to right (OSI Rainbow, maximum intensity top; minimum intensity bottom). (n) FLIR thermography of MD‐III laser positioned for transcutaneous administration to the skin overlying T8 in the cadaveric SD rat post‐DCC injury. Varying power outputs in % of maximum output (800 mW) denoted for each. Mean of *n* = 3, error bars not shown for clarity, laser ON (10 s) and laser OFF (70 s) time points denoted with labeled lines. O: FLIR thermography imaging pre‐ (baseline) and post‐ (60 s) exposure.

To establish the efficacy of a dosing regimen in vivo via implantable and transcutaneous methods, cadaveric modeling was performed to validate the deliverable doses via each method. A method was first developed for delivery of doses within the effective dosing window established in vitro, using an implanted device in a manner suitable for an in vivo murine model (schematic of concept in Figure 3a). A prototype probe was developed using diffusion tip optical components for optogenetics (Figure 3b). The probe was implanted during a post‐mortem procedure to replicate the SCI model (DCC) to be used *in vivo*.^50^, ^53^, ^56^ Power transmission recordings and beam profilometry were again captured at the level of the dorsal column orientated to capture transmission through the laminectomy site (spinal cord removed) (Figure 3c–f). Measurements were taken from *n* = 3 cadavers with implants, using laser power output settings from 50% to 100% in 5% increments (Figure 3c–f). Lower power indices were observed with 100% output, indicating a potential disruption of the fiber or diffuser tip at the highest output settings. Laser output settings of 50%, 55%, and 60% achieved average irradiance values of 13.11, 24.42, and 45.30 mW/cm^2^ respectively.

In vivo, dose selection considered several factors which were likely to compromise PBM delivery efficacy and constitute a wide variety of possible factors which could not be adequately modeled ex vivo, and for which there is no informing literature. For example, during cadaveric modeling, there was considerable variation in transmission dependent on probe positioning or orientation. The above values were measured with the probe optimally placed, which would not be a guarantee post‐implantation after animal movement. Furthermore, incomplete coupling at the ferrule connector could also compromise delivery, and this eventuality was deemed more likely when attaching the source in the in vivo setting. Also, post‐operative bleeding in vivo would attenuate PBM transmission due to the high attenuation of 660 nm light caused by hemoglobin. Further factors were postulated which may also attenuate transcutaneous transmission, such as animal weight gain during a 7‐day treatment regimen, and the effect of oxygenated hemoglobin in vivo. Of the profiled dose delivery settings (13.11, 24.42, and 45.30 mW/cm^2^), 24.42 mW/cm^2^ was selected, in order to sufficiently account for these potential losses. Selection of a higher average irradiance value was precluded by rising peak irradiance values (Figure 3e). Previous work has shown a biphasic response to PBM^59^ and thus it is critical to deliver the correct irradiance at the target site within the effective window, minimizing areas of over‐ or under‐exposure. 24.42 mW/cm^2^ allows the optimal volume of tissue to be irradiated with irradiances within the therapeutic window and also with minimal localized heating.

In further support of this selection, during in vitro modeling, greater consistency and efficacy were observed in dosing at higher (22 and 36 mW/cm^2^) than lower (4 mW/cm^2^) average irradiance values with respect to the optimum (11 mW/cm^2^). Therefore, to account for variability of delivery in cadaveric modeling for both modalities, 24.42 mW/cm^2^ was deemed an appropriate compromise to ensure dose delivery within the effective window on each occasion and across individuals. Besides, had the full 24.42 mW/cm^2^ been transmitted in vivo, this dose is still within the effective range determined by the in vitro experiments above (see Figure 2b).

To validate the safety of the implant, the probe was placed directly above the spinal cord with the tip secured within the laminectomy site (<1 mm distance from cord) with a FLIR camera focused on the cord surface. Regions of interest were recorded from the spinal cord and the probe tip. No discernable heating was recorded at the spinal cord level over a 1‐min exposure, up to 154 mW/cm^2^ (Figure 3g, h). There was transient but negligible probe tip heating at the points of powering on and off the laser source (Figure 3g).

### 3.4 Transcutaneous delivery of PBM to the injured rat spinal cord

After determining the dose of 24.42 mW/cm^2^ for application via the implant, with confirmation that this dose was both achievable and safe, further cadaveric profiling was performed to determine parameters for transcutaneous PBM which would achieve the same average irradiance at the level of the spinal cord. Transcutaneous PBM was applied to the dorsum of a rat cadaver that had undergone post‐mortem SCI modeling (DCC),^50^, ^53^, ^56^ using an MD‐III 800 mW laser coupled to a custom fiber bundle (Figure 3i). Power transmission recordings and beam profilometry were captured at the level of the dorsal column (via anterior approach and corpectomy, with the spinal cord removed) at 50%, 75%, and 100% power outputs (Figure 3j–m). Beam profiles were recorded through the laminectomy site (Figure 3m). This allowed interpolation of the required power output of the laser source to achieve 24.42 mW/cm^2^. Laser power output values were measured to achieve an average irradiance value of 24.42 mW/cm^2^ at the level of the spinal cord for both modalities. For tcPBM, this was a 58.18% power output setting, measured as 229.7 mW output of the source at the skin. For iPBM, this was a 55% power output setting, measured as a 12.8 mW output of the source at the SMA:SMA connector.

Thermography of the skin was performed during irradiation to measure temperature rise and ensure the safety of applying this transcutaneously (Figure 3n, o). This was performed on room temperature cadavers, with a FLIR camera focused on the dorsal skin surface in direct contact with the probe (Figure 3g) during an 80 s treatment protocol (10 s baseline, 60 s laser active period, 10 s post‐treatment observation). The therapeutic level (229.7 mW) led to less than 3°C skin surface heating over the 1 min treatment period. As cadaveric modeling would not replicate heat distribution physiology observed in vivo (via local vasodilatation acting as a vascular “heat sink” via local superficial blood flow), this level was deemed safe and compatible with our stringent concern for animal welfare during in vivo experiments.

### 3.5 PBM promotes early axonal regeneration in vivo

**FIGURE 4:**
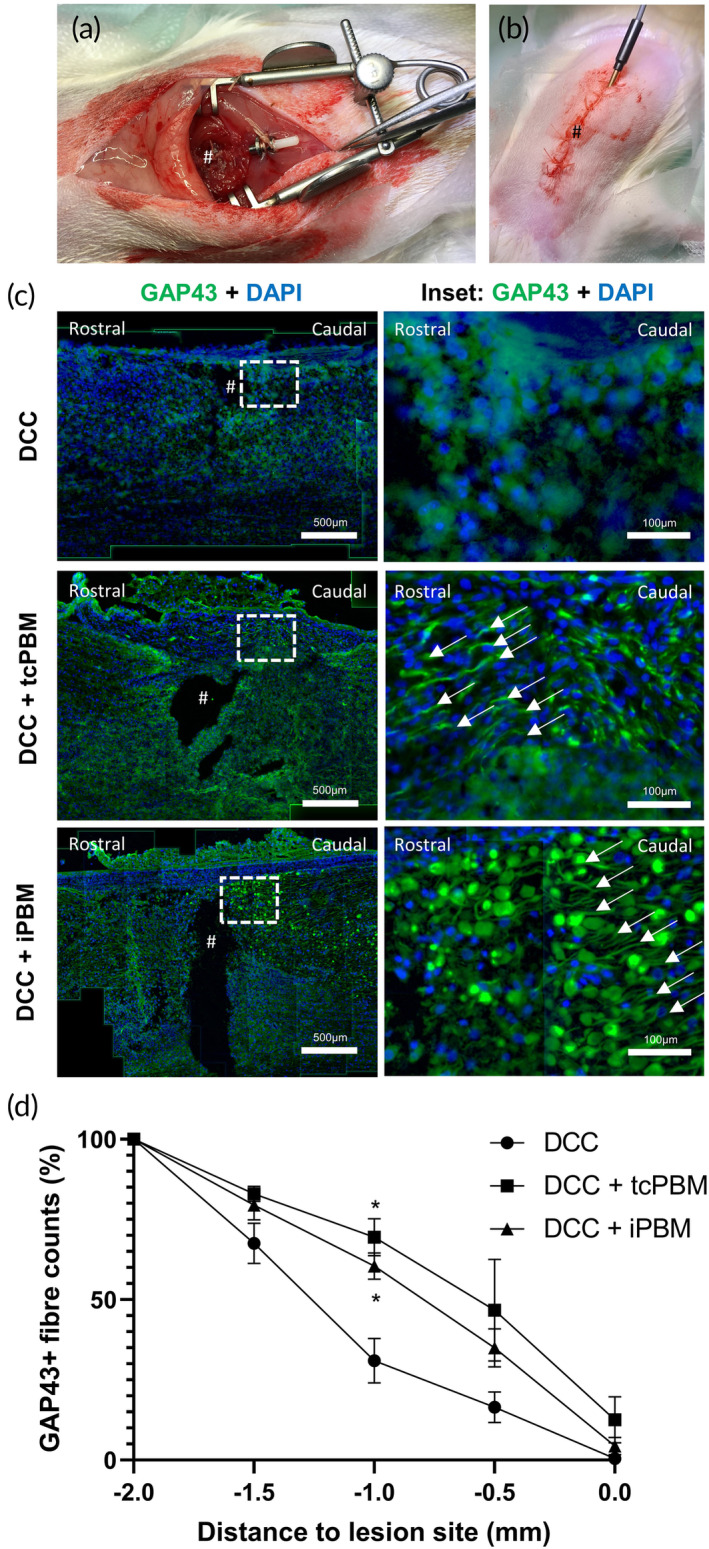
Implantation of a PBM device in vivo increases GAP43 localisation. (a) Photograph of implantation of device, secured to the closed dorsal musculature prior to skin closure, # = lesion epicenter. (b) Photograph depicting implantable PBM system delivering therapy immediately post‐procedure. (c), (d) PBM delivered by transcutaneous (tcPBM) and implanted device (iPBM) promotes early axonal regeneration. A total of 660 nm tcPBM and iPBM were delivered daily for 1 min at 24.4 mW/cm^2^ at the level of the injury site. Injury controls received ambient light transcutaneously for 1 min per day. (c) Immunohistochemistry of sagittal frozen sections through the injury site (labeled #). *n* = 4 per group. (d). Percentage of GAP43 positive fiber counts approaching the epicenter of the injury site from rostral to caudal. *n* = 4 per group.

Implantation in vivo was trialed (Figure 4a, b). Implantation was successful, as were subsequent administrations of intermittent daily doses of PBM. Implants remained in place, functioning and accessible, reliably for 3 days. After this point, increasing animal activity and curiosity, coupled with reducing sensitivity in the postoperative recovery phase, led to device damage and displacement, so experiments using implanted devices were limited to 72 h duration (3× PBM treatments). All experiments used in the subsequent analysis were confirmed as successful *post‐mortem* through dissection and confirmation of an intact, correctly positioned, and functioning probe after removal at termination of the experiment.

**FIGURE 5:**
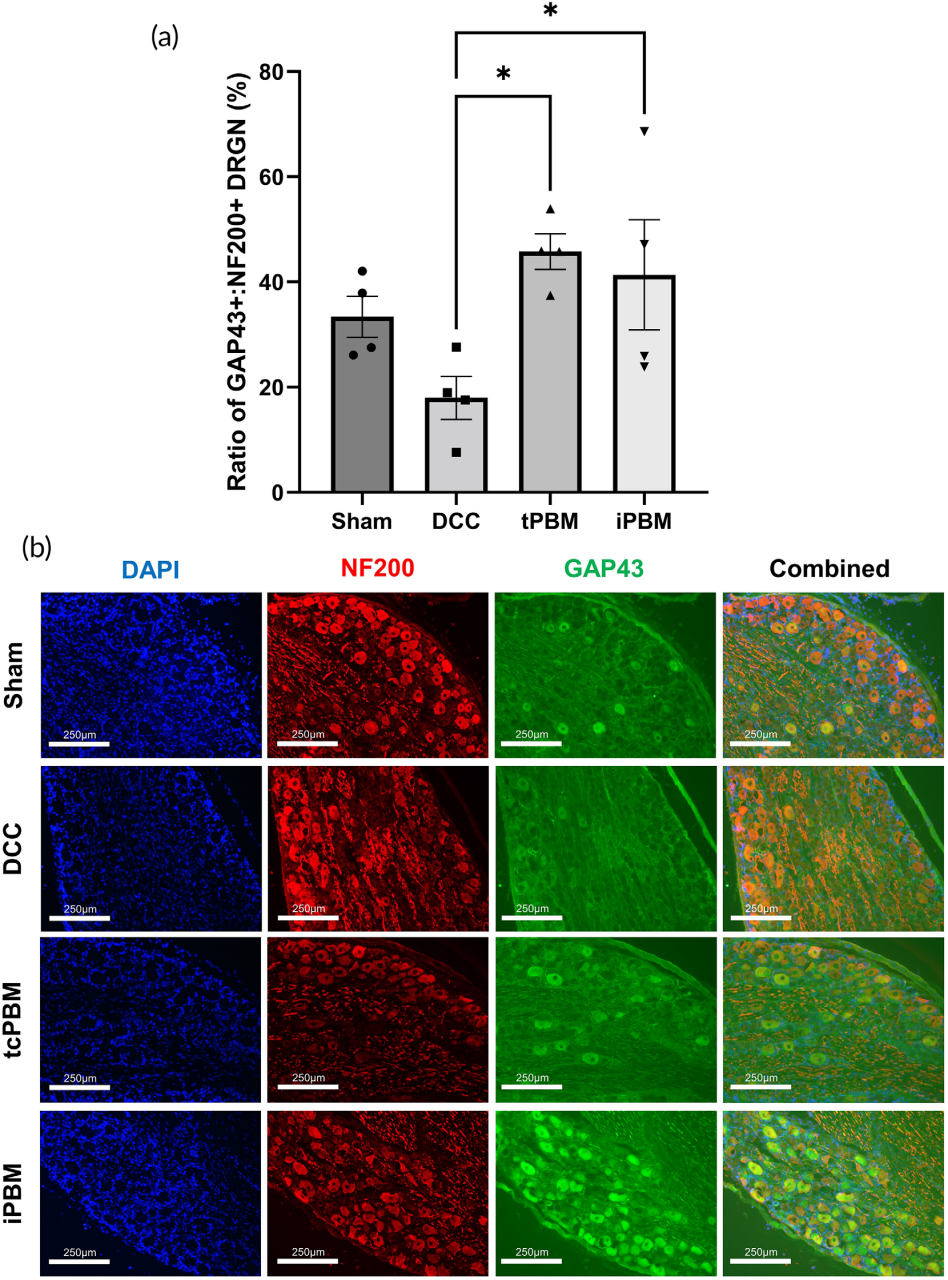
PBM delivered by transcutaneous (tcPBM) and implanted device (iPBM) promote early upregulation of GAP43 expression within dorsal root ganglion neurons. A total of 660 nm tcPBM and iPBM were delivered daily for 1 min at 24.4 mW/cm^2^ at the level of the injury site. Injury controls received ambient light transcutaneously for 1 min per day, with termination 3 days post‐injury. (a) Percentage of GAP43 positive DRG (% of NF200 positive cells co‐expressing GAP43). *n* = 4 per group, (b) Representative immunofluorescence images from each group at 10× magnification, from which results in (a) are taken. *n* = 4 per group.

Implantable (iPBM) and transcutaneous (tcPBM) were directly compared at 3 days post‐injury (dpi) after application of 24 mW/cm^2^ daily for 1 min, commencing 15 min post‐injury. As an early marker of initiation of neurorestorative processes, regeneration‐associated protein (RAP) localization within axons (e.g., growth associated protein‐43 (GAP43))^50^, ^56^, ^60^ approaching the injury site were evaluated after tcPBM, iPBM, and sham therapy (DCC) (Figure 4c, d). Increased presence of axons 1 mm caudal to the injury site reached statistical significance for both tcPBM (*) and iPBM (*) compared with DCC (Figure 4d). This correlated with increased expression within DRGN in L4/5 DRG after both methods of PBM therapy, suggesting RAP activation within the cell body of neurons affected by injury (Figure 5a, b). Analysis of DCC DRG found that 18.0% of DRGN cells expressing NF200 co‐expressed GAP43. This increased to 45.8% ± 3.4 and 41.4% ± 10.5 of DRGN after tcPBM and iPBM respectively (both *). This was further confirmed through correlation with the same effect observed in pilot experiments after 6 dpi with 6 daily applications of tcPBM (Supplementary Figure 1).

### 3.6 PBM promotes axonal regeneration and functional recovery

**FIGURE 6:**
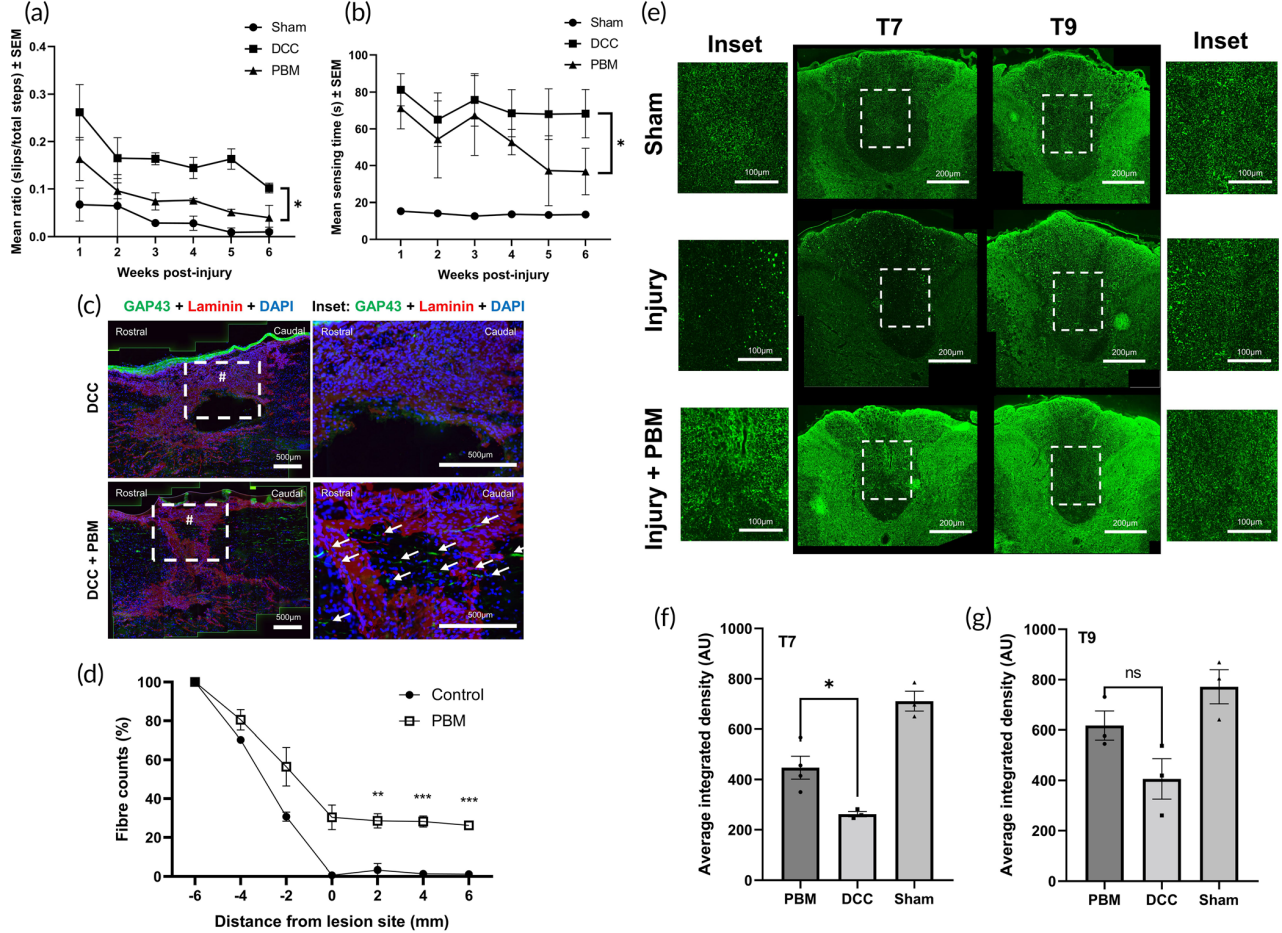
GAP43^+^ axons traverse the glial scar at 6 weeks post‐SCI with PBM therapy. (a), (b) Functional recovery at 6 weeks post‐injury improved with tcPBM. (c) Horizontal ladder crossing test, and (d) mean sensing time for the hind paw tape sensing and removal test. *n* = 4–6 per group. (c) Immunofluorescence for GAP43 (green), laminin (red), and DAPI (blue) of T8 lesion site (taken from middle 15 μm parasagittal section) for injured control with sham therapy (DCC) and injury + PBM (DCC + PBM). # = lesion site, ↙ = GAP43^+^ axon. (d) Axon counts from 4 mm caudal to 6 mm rostral to center of SCI lesion site in control (DCC and sham therapy) and injured specimens treated with PBM. *N* = 4 per group. (e)–(g) tcPBM increases NF200 immunoreactivity rostral to the lesion site. (e) Axial sections through T7 (rostral to lesion site) and T9 (caudal to lesion site) underwent immunostaining for NF200 (green). (f) Intensity of immunofluorescence in the entire dorsal fasciculus at T7 and (g) T9 were analyzed and compared between the three groups. PBM = transcutaneous PBM, DCC = Injury (sham therapy control). *n* = 4 per group. Sham = sham surgery (no injury). AU = arbitrary units.

In further experiments, tcPBM was applied daily for 7 days, with weekly functional assessments of locomotor and sensory recovery for 6 weeks. Functional recovery was demonstrated with a statistically significant improvement in locomotor recovery based on the horizontal ladder crossing test with tcPBM (*p* = 0.012) (Figure 6a), and in improvement of mean sensing time on the tape removal test (*p* = 0.011) (Figure 6b). At 6 weeks, a 1 cm segment of spinal cord at T8 was taken and sliced in sagittal sections. The central slide (two slices through the center of the lesion site) was used to assess axonal regeneration. tcPBM was associated with a significant increase in the number of GAP43^+^ axons 4 and 6 mm rostral to the injury site (Figure 6c, d) (30.9% ± 5.0 and 29.1% ± 4.8 respectively) with clear visualization of axons traversing the laminin rich glial scar (Figure 6c bottom right panel). Intensity of NF200 immunofluorescence in the dorsal fasciculus in axial sections from T7 (rostral) and T9 (caudal) were also analyzed (Figure 6e). This demonstrated a statistically significant increase in average integrated density rostral to the lesion site (T7) in specimens exposed to tcPBM. There was a modest and not statistically significant effect of tcPBM within the T9 segment.

### 3.7 PBM reduces cavitation and glial scar deposition

**FIGURE 7:**
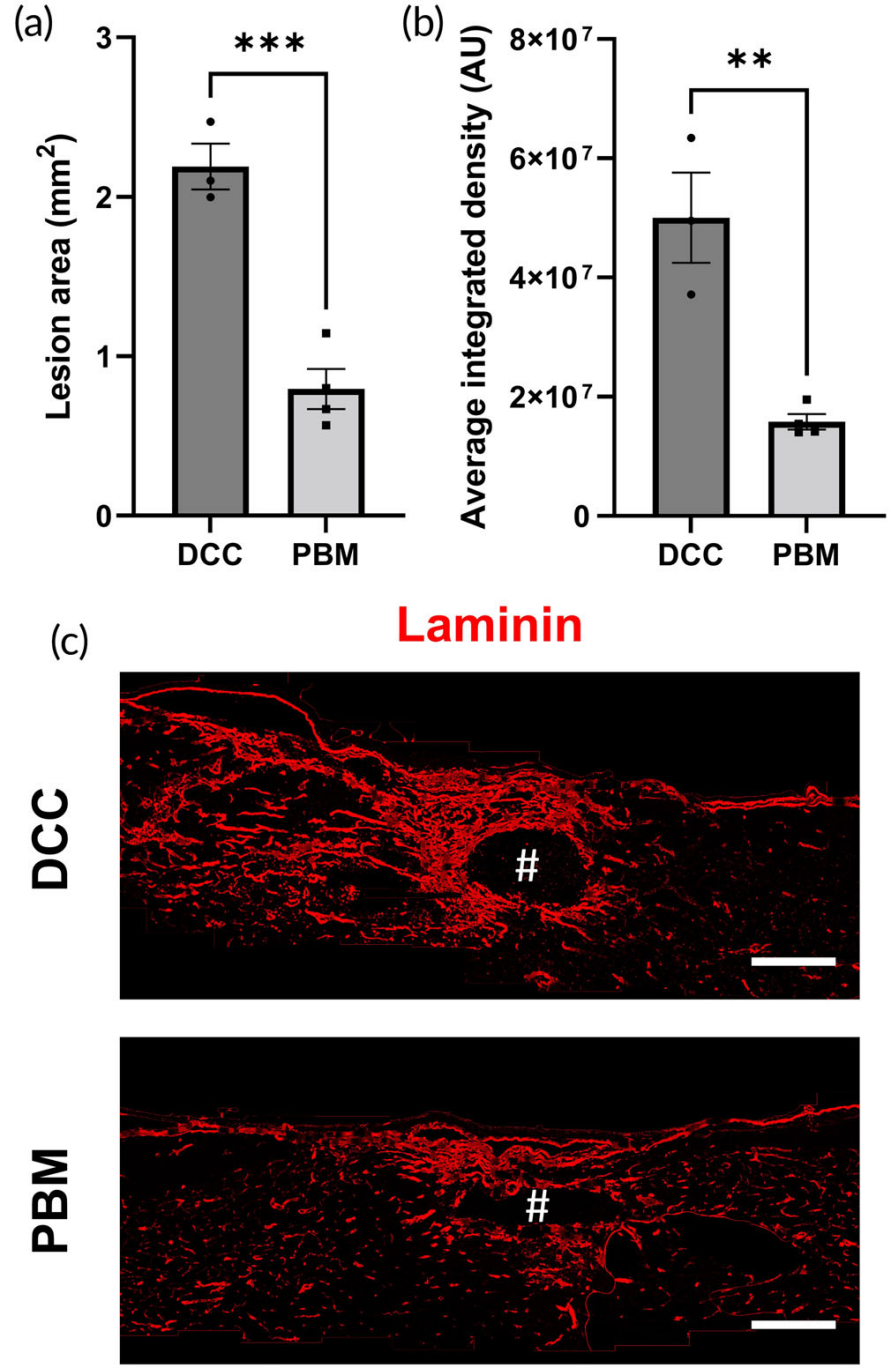
tcPBM is associated with reduced lesion size and reduced laminin immunoreactivity. (a) Sagittal lesion area through the middle of the lesion site was significantly reduced in specimens exposed to tcPBM. (b) tcPBM was associated with lower immunoreactivity after anti‐laminin staining and immunofluorescence. (c) representative images from DCC (injury only with sham therapy) and tcPBM therapy. Scale bars = 500 μm.

The total size of the lesion site (based on extent of cavitation and laminin immunofluorescence) and the intensity of immunofluorescence for laminin staining within this region were compared in sham treatment and tcPBM treatment at 6 weeks post‐injury (Figure 7). The central two slices through the lesion site were used to assess lesion size (Figure 7c). Lesion size after tcPBM treatment reduced in comparison with control (sham‐treated) specimens from 2.19 mm^2^ ± 0.14 (control) to 0.80 mm^2^ ± 0.13 (tcPBM) (***) (Figure 7a). This corresponded to a reduction in immunofluorescent expression of laminin within the glial scar by greater than 50% (**) (Figure 7b).

## 4 DISCUSSION

Here we have demonstrated that 660 nm PBM significantly improves functional and histological outcomes after SCI, with improvements in sensory and locomotor recovery, correlating with increased axonal regeneration, reduced glial scarring, and reduced spinal cord cavity size. Furthermore, we demonstrate that early post‐injury activation of RAPs (e.g., GAP43) can be achieved through direct delivery of PBM via a novel implant method for small mammal PBM therapy, with equivalent early beneficial effects to tcPBM. Through dosimetry in cadaveric studies, we have validated that our two methods deliver equivalent PBM doses to the SCI site, supporting the hypothesis that iPBM achieves similar therapeutic benefits to tcPBM. Whilst delivery of average irradiance values of 24.42 mW/cm^2^ at the level of the spinal cord is feasible in rodents, the thickness of overlying tissue in humans renders this impossible without causing destruction of surface tissue through the use of high transcutaneous power outputs. The implication is that iPBM is a more readily translatable method of application of effective doses of PBM to deep anatomical structures, such as the spinal cord. Demonstration of non‐inferior efficacy to tcPBM supports onward development of implantable devices to achieve these proven therapeutic levels of PBM at the plane of the target tissue.

The dose regimen used in the in vivo study described here is within the broad range of effective doses described in the previous literature.^26^, ^29^, ^30^, ^31^, ^32^, ^33^, ^34^, ^35^, ^36^, ^37^, ^38^ Extrapolated from heterogenous dosimetry reporting, prior studies describing the efficacy of PBM in SCI have utilized average irradiances at between 0.2 and 250 mW/cm^2^, with a range of fluences per treatment of 0.03–95.34 J/cm^2^ using either wavelengths from 660/780/810/850 nm regions.^26^, ^29^, ^30^, ^31^, ^32^, ^33^, ^34^, ^35^, ^36^, ^37^, ^38^ Accordingly, the duration of treatment has also varied, with dose durations varying from 45 and 3000 s. Such broad dosing regimens are problematic for the field of PBM, and development and application of effective technology. Validation of effect is further complicated by variability in transmission through tissue, which is dependent on a broad range of factors, such as superficial tissue thickness and pigmentation. As we have described previously, accurate measurement and reporting are required. Further to this, implantable devices with better line of sight to the injured tissue provides much greater opportunity for accurate and standardized dose delivery which we propose will produce greater effects.

Timing of the first dose has also varied, though the majority have commenced therapy within 30 min of injury. Duration of the course of therapy have also varied significantly in previous work, ranging from 3 to 21 days amongst those demonstrating therapeutic effect. It is notable that Giacci et al. found no positive effect when delivering therapy daily for 28 days post‐injury.^61^ In relation to work in applying PBM after TBI, it has also been noted that longer courses of treatment are less effective.^17^ This effect may be attributable to the therapeutic benefit of PBM mitigating neuroinflammation, which is favorable only within the acute and sub‐acute phases of injury. This study utilized 1.44 J/cm^2^, delivered with an average irradiance of 24 mW/cm^2^ for 60 s per day, with a 7 day course commenced 15 min post‐injury (3 days only at 3 dpi endpoint).

To our knowledge, this is the first report comparing the efficacy of delivering PBM via transcutaneous versus implantable methods in SCI, with matched doses at a tissular level. Few previous works have used direct‐to‐cord methods of PBM application: Ando et al. (2013) applied PBM directly via daily surgery to access the spinal cord for five consecutive days after injury in their rat model, demonstrating improvement in functional outcomes.^29^ Two studies from the Wang group have used a direct diffusion fiber orientated over the spinal cord in a piglet model, though the outcome measures used in these studies sought principally to demonstrate the safety of delivering 200–300 mW outputs directly over the cord.^48^, ^49^

The validation of the comparative efficacy of implantable versus transcutaneous administration is of translational relevance. Direct application methods of PBM delivery have been more extensively described in the context of models of Parkinson's disease,^44^, ^45^, ^47^ where they have been demonstrated to be safe and effective. Further work in this area has also demonstrated that some efficacy in preventing neurodegeneration in Parkinson's disease can be achieved through remote administration of PBM (to a remote anatomical region to the pathological area, i.e. the femur in CNS degeneration).^42^, ^43^, ^44^, ^45^, ^46^ Whilst direct application methods have produced most benefit, the efficacy of remote application raises the possibility of a systemic activation of advantageous biological processes that may be involved in the efficacy of transcutaneous application.^42^, ^43^, ^44^, ^45^, ^46^, ^47^, ^62^ Here, spinal cord level average irradiance was controlled between groups, though the tcPBM arm inevitably received greater overall exposure to PBM due to transmittance through (and absorbance by) superficial tissues (skin, muscle, vasculature, bone). This may explain the marginal (and statistically nonsignificant) differences in early RAP activation between tcPBM and iPBM, though this could equally be attributed to minor deleterious or pro‐inflammatory effects of an indwelling implant. Inclusion of remote application of PBM as a comparative treatment group in future studies may advance the understanding of the contribution of activation of systemic processes in the therapeutic benefits of PBM in SCI.

In the wider literature, neuroinflammation is increasingly recognized as a key mediator of PBM in neurotrauma. Recent work has identified the effect of PBM on reducing polarization to the neurotoxic M1 phenotype for macrophages/microglia,^34^, ^35^, ^36^ (by inhibition of STAT3 expression via increased expression of miR‐330‐5p; and activity in the Notch1‐HIF‐1α/NF‐κB pathway) and the A1 phenotype for astrocytes.^40^ There has also been recent identification of the effect of PBM reducing neuronal mitochondrial fission imbalance after SCI. Together, these further support a mixed effect of PBM on both neuronal and nonneuronal cell populations.^41^

There are some limitations to consider in this work. The effect of the implant itself has not been studied directly, and there may have been an immune response to the presence of the indwelling probe. With extended duration, this may result in a localized fibrotic reaction which may mitigate the potential for benefit. Equally, it is possible that the presence of the probe alone accounts for some of the therapeutic benefit of the implant, independently from the PBM treatment, which has not been studied through use of a group receiving an implant without PBM application. This should be the subject of future research. Similarly, concerns about animal welfare precluded a longer duration of study with the implant in situ, and subsequent work should include longer timepoints. Furthermore, utilizing ex vivo cadaveric models inherently limits the accuracy of dosimetry measurements, as the in vivo setting will differ considerably due to a number of effects including those of blood flow, oxygenated hemoglobin, and progressive fibrosis. In vivo measurements are the optimal to ensure accuracy, though this was not technically achievable in this injury model.

The decision to implant and deliver the first PBM dose immediately after wound closure and within minutes post‐injury was to optimize efficacy based on the hypothesis that PBM mitigates the early damaging effects of neuroinflammation. It is currently not known whether these parameters for PBM delivery are effective if commenced at a later time point. Discerning a therapeutic window within which therapy must be commenced to achieve efficacy will be the subject of further study to ensure translational relevance, given that timing of surgical intervention in SCI is typically at least 4 h post‐injury, and may be up to 24/48 h. Efficacy of PBM in reducing neuropathic pain have proven effective after sciatic nerve injury when commencing therapy 1 day^63^ and 1 week^64^ post‐injury, though the mechanisms involved differ considerably. Another possibility for applications in sub‐acute/chronic settings is utilizing PBM to augment other therapeutic interventions. A growing area is the use of PBM to promote functional differentiation of stem cells toward neural repair, which has proven successful in pre‐clinical testing for SCI and other neural injury models.^65^, ^66^, ^67^

The failure of the NEST‐3 trial to show significant benefit to patients of PBM after ischaemic stroke has highlighted the necessity for rigorous pre‐clinical optimization of PBM therapeutic protocols prior to clinical trials.^68^, ^69^ Given the breadth of possible parameters for PBM treatment, more extensive optimization is required than in comparable studies using pharmaceutical treatments. Delivery modalities should be considered alongside wavelength and dosimetry variables as a key feature of PBM which can considerably affect its efficacy in treating the injured CNS.

In this study, we have developed a method for PBM dose optimization for the neuroprotection of sensory neurons and a novel approach for the direct application of PBM to the spinal cord, with dosimetry validation of this and transcutaneous PBM in a cadaveric model. Comparison of these two application methods has demonstrated equivalence of PBM‐mediated activation of RAPs after SCI through invasive or noninvasive delivery paradigms, which correlates with significant improvements in functional recovery, axonal regeneration, and lesion size after SCI in a rat model. Administration of PBM via direct application to injured tissue should be further explored as a translational approach to developing future PBM‐based treatments for SCI.

## AUTHOR CONTRIBUTIONS

**Andrew R. Stevens:** Conceptualization; data curation; formal analysis; funding acquisition; investigation; methodology; project administration; validation; writing – original draft; writing – review and editing. **Mohammed Hadis:** Conceptualization; funding acquisition; investigation; methodology; resources; software; supervision; validation; writing – review and editing. **Alice Phillips:** Data curation; investigation. **Abhinav Thareja:** Data curation; investigation; validation. **Michael Milward:** Conceptualization; funding acquisition; resources; supervision; writing – review and editing. **Antonio Belli:** Conceptualization; funding acquisition; resources; supervision; writing – review and editing. **William Palin:** Conceptualization; funding acquisition; methodology; resources; supervision; writing – review and editing. **David J. Davies:** Conceptualization; funding acquisition; methodology; writing – review and editing. **Zubair Ahmed:** Conceptualization; data curation; formal analysis; funding acquisition; investigation; methodology; project administration; resources; software; supervision; validation; visualization; writing – review and editing.

## FUNDING INFORMATION

This study received funding from the Wellcome Trust, grant no. 213458/Z/18/Z and Royal College of Surgeons of England Surgical Research Fellowship. A.R.S. also received funding support from the NIHR Surgical Reconstruction and Microbiology Research Centre (SRMRC).

## CONFLICT OF INTEREST STATEMENT

Members of the authorship have submitted a patent pending application (DD, MH, WP, ARS and ZA) relating to the invasive delivery of PBM (UK Patent App. No. 2006201.4; US Patent App. 17/922, 157, 2023). There are no other competing interests to declare, including those relating to employment, consultancy, other patents, or products in development.

## Supporting information


**Supplementary Figure 1.** Top: Increased expression of GAP‐43^+^ axons within the dorsal column after exposure to PBM therapy versus sham‐treated control at day 7 post‐injury. *n* = 2 per group. DC = dorsal column crush; PBM = photobiomodulation treatment (transcutaneous 22 mW/cm^2^ (at lesion site), 1 min per day, 6‐day treatment course). R = rostral, C = caudal, # indicates lesion site. Bottom: Increased expression of GAP‐43^+^ DRGN after exposure to PBM therapy versus untreated control at day 7 post‐injury. n = 2 per group. DC = dorsal column crush; PBM = photobiomodulation treatment (transcutaneous 22 mW/cm^2^ (at lesion site), 1 min per day, 6 day treatment course).

**Supplementary Table 1.** Antibody clones used.

## Data Availability

Data supporting this study are available on reasonable request made to the corresponding author.
