# Supplementary figures and images for: Implantable and transcutaneous photobiomodulation promote neuroregeneration and recovery of lost function after spinal cord injury
Source: Bioeng Transl Med. 2024 Apr 25;9(6):e10674. doi: 10.1002/btm2.10674 (PMC11558183; doi:10.1002/btm2.10674)

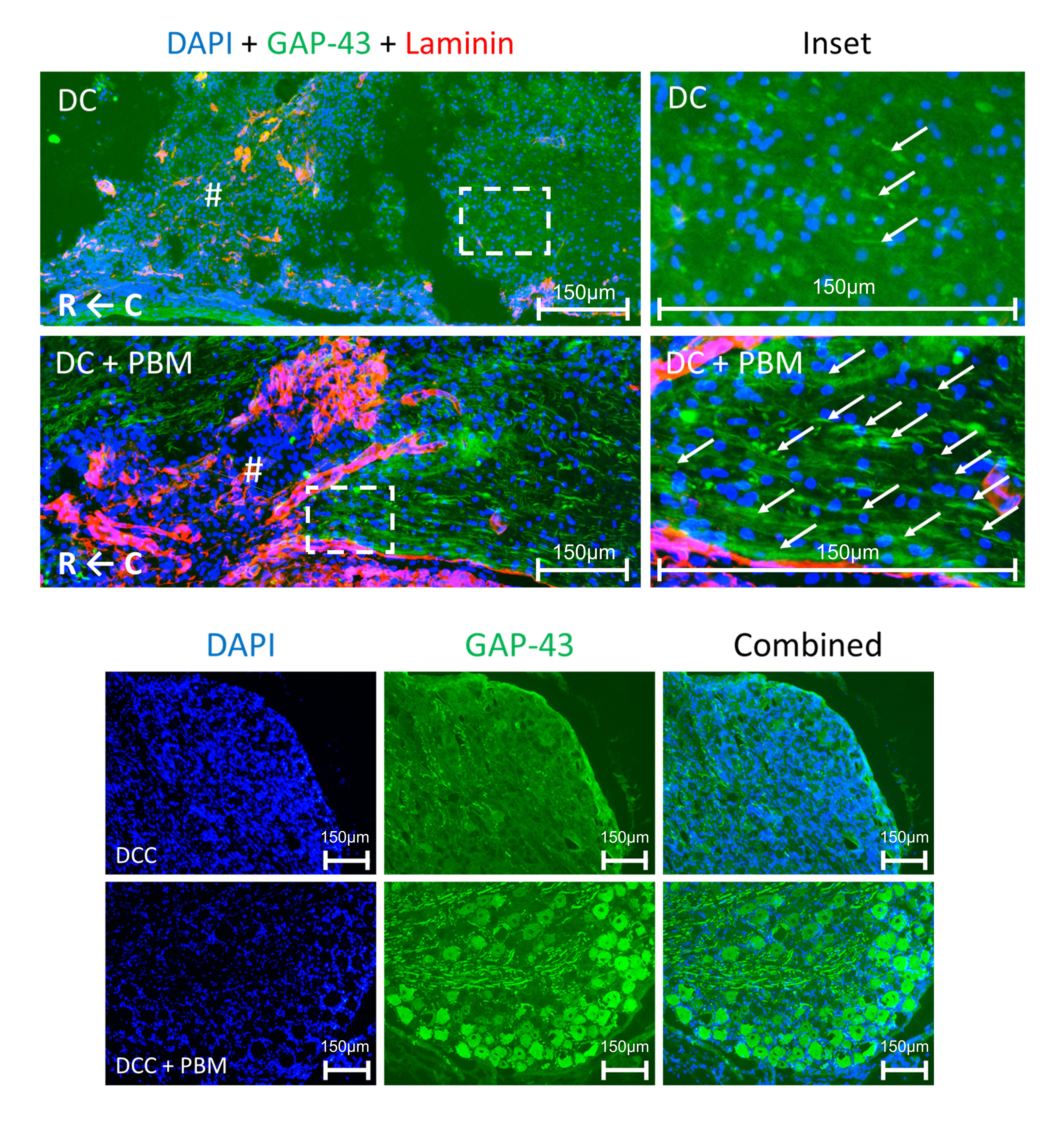

Supplement: Supplementary file 1 — Supplementary Figure 1. Top: Increased expression of GAP‐43+ axons within the dorsal column after exposure to PBM therapy versus sham‐treated control at day 7 post‐injury. n = 2 per group. DC = dorsal column crush; PBM = photobiomodulation treatment (transcutaneous 22 mW/cm2 (at lesion site), 1 min per day, 6‐day treatment course). R = rostral, C = caudal, # indicates lesion site. Bottom: Increased expression of GAP‐43+ DRGN after exposure to PBM therapy versus untreated control at day 7 post‐injury. n = 2 per group. DC = dorsal column crush; PBM = photobiomodulation treatment (transcutaneous 22 mW/cm2 (at lesion site), 1 min per day, 6 day treatment course). [file BTM2-9-e10674-s002.JPG]
