# Supplementary material for: Implantable and transcutaneous photobiomodulation promote neuroregeneration and recovery of lost function after spinal cord injury
Source: Bioeng Transl Med. 2024 Apr 25;9(6):e10674. doi: 10.1002/btm2.10674 (PMC11558183; doi:10.1002/btm2.10674)
Supplement: Supplementary file 2 — Supplementary Table 1. Antibody clones used. [file BTM2-9-e10674-s001.docx]

Supplementary Table 1. Antibody clones used.

| Antigen | Dilution | Supplier | Catalogue No. |
| --- | --- | --- | --- |
| GAP43 | 1:400 | Fisher Scientific, Loughborough, UK | 33-5000 |
| Laminin | 1:400 | Sigma, Poole, UK | L9393 |
| NF200 | 1:400 | Sigma, Poole, UK | N4142 |
| Goat anti-mouse IgG (Alexa Fluor 488) | 1:400 | Fisher Scientific, Loughborough, UK | A-11011 |
| Goat anti-rabbit IgG (Alexa Fluor 594) | 1:400 | Fisher Scientific, Loughborough, UK | A-11012 |
| Annexin-V (labelled with iFluor 594) | As per manufacturer | Abcam, Cambridge, UK | ab219918 |
